# Improvement in the Accuracy of the Postclassification of Land Use and Land Cover Using Landsat 8 Data Based on the Majority of Segment-Based Filtering Approach

**DOI:** 10.1155/2021/6658818

**Published:** 2021-03-19

**Authors:** Fajar Yulianto, Gatot Nugroho, Galdita Aruba Chulafak, Suwarsono Suwarsono

**Affiliations:** Remote Sensing Applications Center, Indonesian National Institute of Aeronautics and Space (LAPAN), Jl. Kalisari No. 8, Pekayon, Pasar Rebo, Jakarta 13710, Indonesia

## Abstract

Improvement in the accuracy of the postclassification of land use and land cover (LULC) is important to fulfil the need for the rapid mapping of LULC that can describe the changing conditions of phenomena resulting from interactions between humans and the environment. This study proposes the majority of segment-based filtering (MaSegFil) as an approach that can be used for spatial filters of supervised digital classification results. Three digital classification approaches, namely, maximum likelihood (ML), random forest (RF), and the support vector machine (SVM), were applied to test the improvement in the accuracy of LULC postclassification using the MaSegFil approach, based on annual cloud-free Landsat 8 satellite imagery data from 2019. The results of the accuracy assessment for the ML, RF, and SVM classifications before implementing the MaSegFil approach were 73.6%, 77.7%, and 77.5%, respectively. In addition, after using this approach, which was able to reduce pixel noise from the results of the ML, RF, and SVM classifications, there were increases in the accuracy of 81.7%, 85.2%, and 84.3%, respectively. Furthermore, the method that has the best accuracy RF classifier was applied to several national priority watershed locations in Indonesia. The results show that the use of the MaSegFil approach implemented on these watersheds to classify LULC had a variation in overall accuracy ranging from 83.28% to 89.76% and an accuracy improvement of 6.41% to 15.83%.

## 1. Introduction

Land use and land cover (LULC) is a key driver of environmental change and can describe the conditions of changing phenomena resulting from human interaction with the environment [1]. It is important information that has implications for the sustainable use of resources in watershed management activities, as it generally reflects irreparable degradation or loss of land and water resources [2]. The utilisation of land, space, and resources for settlement, agriculture, tourism, industry, and transportation will continue to increase for some time to come [3, 4]. Remote sensing data can be used for the analysis, monitoring, mapping, and classification of LULC information. Its availability provides a choice of resolution variations (spectral, spatial, radiometric, and temporal) to detect land changes on the earth's surface, by comparing current multitemporal conditions with those of previous years [5–8].

The object classification of LULC on the earth's surface based on remote sensing data can be processed with two digital classification methods, namely, supervised and unsupervised classification [9]. The supervised version involves the classification of objects based on training sample input from object classes that appear on satellite images, which are then run using an algorithm to generate LULC information. These are maximum likelihood [10–14], minimum distance [15], convolutional neural networks [16], decision tree [17], random forest [18–20], support vector machine [21, 22], and K-nearest neighbor [23, 24]. On the other hand, unsupervised classification involves data processing that can be conducted based on cluster pixel values in a satellite image (spectral, temporal, and spatial information) into value classes, which are run using a clustering algorithm; these are iterative self-organizing data analysis [3, 25] and K-means clustering [26, 27].

There are several limitations and problems with digital classification results; for example, pixel noise can affect the spatial accuracy and quality of LULC information. In the postclassification stage, LULC can be produced using a spatial filter to reduce this noise and to obtain better results. These include the mean, standard median, adaptive wiener, Gaussian, adaptive median filters [28–30], majority filter [31, 32], and object filter based on the topology and feature approach [33]. With the use of spatial filters, pixel noise is sometimes still left. To overcome such an obstacle, majority segment-based filtering (MaSegFil) is proposed in this study as a spatial filter stage in the postclassification, used to classify objects on the earth's surface based on the digital classification results. The purpose of using the MaSegFil approach is to reduce pixel noise from these results and to obtain better information on object classification results.

In this study, we first analyse whether incorporating the MaSegFil approach at the postclassification stage improves the accuracy of the digital classification and reduces the resulting pixel noise. Furthermore, accuracy assessment based on reference data is used to compare the postclassification results before and after the implementation of the MaSegFil approach. The principles of this approach are that (a) the area boundary of the class of objects on the earth's surface seen in the satellite image data will be separated by patterns based on the segment mean shift process (segmentation process); (b) the results of the digital classification that has been made are used as inputs to fill in the attribute class of objects in each area segment that has been separated, based on its segmentation pattern; (c) the extraction of the attribute value of the digital classification results in each area segment (object segmentation results) is made by taking the majority value, or the most dominant object, and using it as the spatial filter in the area based on the zonal statistics spatial analyst calculation with the type majority; and (d) the final results of the calculation are used as the output of object classification at the postclassification stage.

### 1.1. Study Area

**Figure 1:**
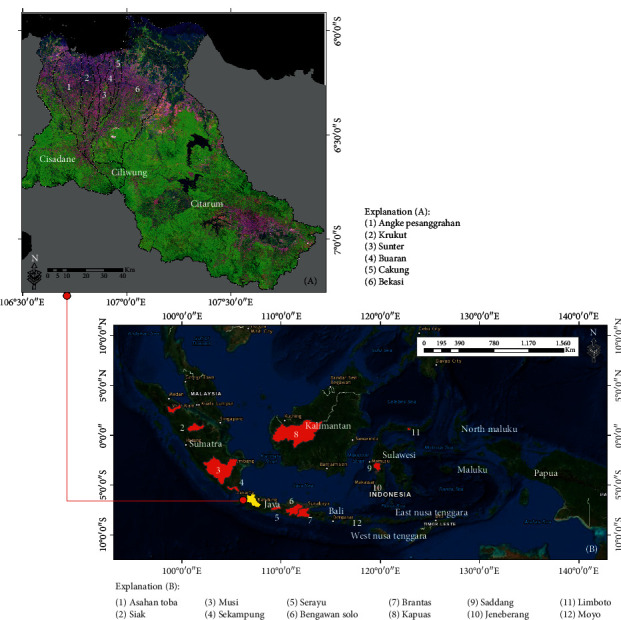
The 15 national priority watersheds in Indonesia: (a) test case study areas in the Citarum, Ciliwung, and Cisadane watersheds; (b) another priority watershed location for the implementation of the method proposed.

To be able to understand and implement the proposed approach method, we chose to study areas in the Citarum, Ciliwung, and Cisadane watersheds, which are part of the 15 national priority watersheds in Indonesia, located in the provinces of Banten, Jakarta, and West Java, Indonesia (on Java island) (Figure 1(a)). The study area has a wide variety of LULC classes that can be used as samples, representing some of the objects that will be classified based on the remote sensing satellite imagery. Furthermore, the proposed method was also applied to several other locations, including the 12 national priority watersheds in Indonesia, which represent the characteristic variations of LULC and are located on the islands of Sumatra (Asahan Toba, Siak, Musi, and Sekampung), Java (Serayu, Bengawan Solo, and Brantas), Kalimantan (Kapuas), Sulawesi (Saddang, Jeneberang, and Limboto), and West Nusa Tenggara (Moyo) (Figure 1(b)).

## 2. Methods

**Figure 2:**
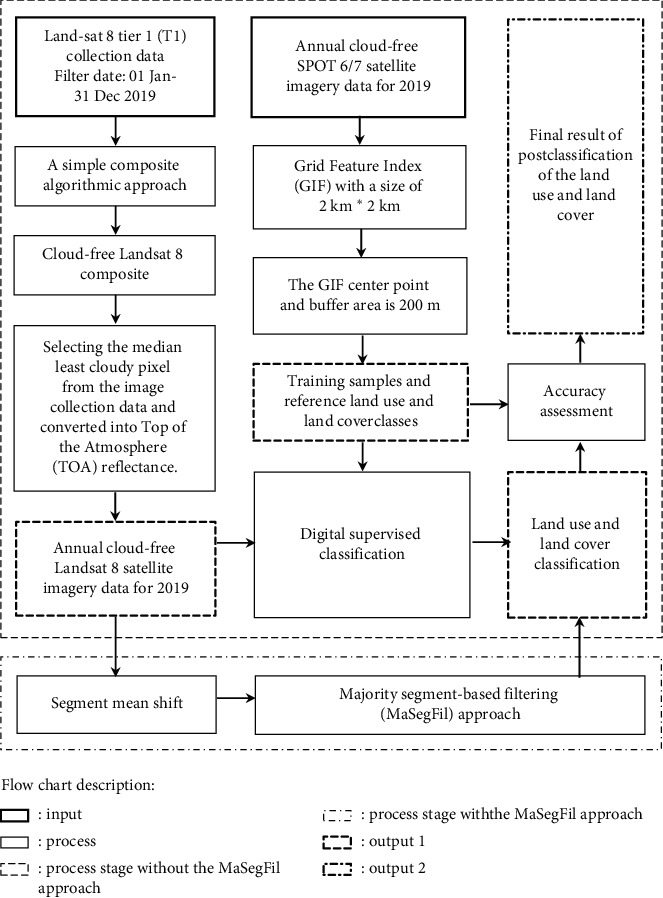
Study flowchart.

The proposed method used in this study is presented in Figure 2, comprising several sections consisting of data availability, LULC classification, postclassification, accuracy assessment, and comparison of classification performance results.

### 2.1. Data Availability

Objects on the earth's surface covered by clouds are a major problem in the use of optical image data. This can be overcome by creating cloud-free satellite imagery data annually. In this study, data processing was performed using the Google Earth Engine (GEE) platform. The input data were obtained from the USGS Landsat 8 Surface Reflectance Tier 1 data collection. This dataset comprises the atmospherically corrected surface reflectance from the Landsat 8 OLI/TIRS sensors, which is based on the Landsat Ecosystem Disturbance Adaptive Processing System (LaSRC). The various stages of the process consist of cloud, shadow, water, and snow mask, which are produced using CFMASK [34–36]. Information and detailed technical explanations can be accessed at https://developers.google.com/earth-engine/datasets/catalog/LANDSAT_LC08_C01_T1_SR. Filter dates are needed to determine the date range selection to get the annual Landsat 8 in 2019. In this case, the filter dates were limited to between 1 January and 31 December 2019. Furthermore, high-resolution imagery mosaic SPOT 6/7 from 2019, which can be obtained from the Remote Sensing Technology and Data Center, LAPAN, was used as an input training sample and reference data for assessing the accuracy of the LULC classification results produced by the study.

### 2.2. LULC Classification Approach

In this study, we used digital classification to reproduce the LULC information. The classification approaches included maximum likelihood (ML), random forest (RF), and support vector machine (SVM) classifiers. Furthermore, LULC classification resulting from the digital classifications was evaluated by assessing the accuracy based on the reference data. This was done to determine the optimal classification approach to classifying LULC in the study area. Eleven LULC classes were employed, which refer to the National Standardization Agency for Indonesia [37]; detailed related information is presented in Table 1.

**Figure 3:**
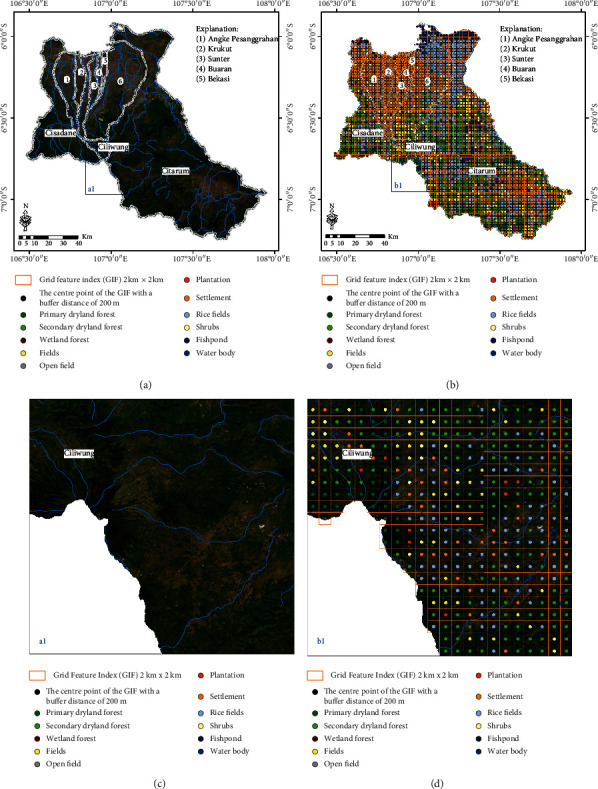
Determination of training samples and reference map of the study area: (a, c) annual mosaic of SPOT 6/7 images in 2019; (b) compilation of the Grid Feature Index (GIF) with dimensions of 2 km × 2 km is carried out to determine training samples and LULC. references; (d) centre point of each GIF block buffered with a distance of 200 m is used as the location for training samples and references from LULC classes.

A training sample and reference maps were produced referring to the annual mosaic image data of SPOT 6/7 2019 images obtained from the Remote Sensing and Technology Data Center, LAPAN. Arrangement of the Grid Feature Index (GIF) with a size of 2 km × 2 km was made to determine the training sample and reference data more systematically, based on visual interpretation. Furthermore, the centre point of each GIF block, which had been buffered with a distance of 200 m to obtain the polygon area, was used as a location for the training samples and also as a reference for LULC classes in the study area (Figure 3).

### 2.3. Maximum Likelihood (ML) Classifier

The ML classifier is a digitally supervised classification approach that applies the Gaussian threshold in several class signatures to assign every pixel class. The approach assumes that the probability of the model class distribution is multivariate normal. In detail, the maximum likelihood classifier formulation is presented in equations (1) and (2) [1, 2, 7, 38–40]:

(1) $$\begin{array}{c} G_{i}\left(x\right)=\ln\;p\left(\omega_{i}\right)-\frac{1}{2}\ln\left|\Sigma_{i}\right|-\frac{1}{2}\left(x-m_{i}\right)\sum_{i}^{-1}\left(x-m_{i}\right). \end{array}$$

Therefore,

(2) $$\begin{array}{c} x\in \omega_{i},\;\text{if }g_{i}\left(x\right)>g_{j}\left(x\right)\text{ for all }j\neq i, \end{array}$$

where *G*_*i*_(*x*) is the discriminant function in the ML algorithm; *ω*_*i*_ is the class (where *i*=1,…, *n*); and *M* is the total number of classes. *x* is a pixel in the *n*-dimensional vector (where *n* is the number of bands on the satellite image is used). *p*(*ω*_*i*_) is the true class opportunities, in *ω*_*i*_ for pixel positions *x*; |Σ_*i*_| is the decisive determinant of the covariance matrix of data in the *ω*_*i*_ class; Σ_*i*_ is the inverse covariance matrix of the data in the *ω*_*i*_ class; and *m*_*i*_ is a vector average.

### 2.4. Random Forest (RF) Classifier

The RF classifier is a digitally supervised classification approach, consisting of a combination of tree classifiers. Each classifier is created using a random vector sampled independently of the input vector. Furthermore, each tree cast will provide calculations on the most dominant class unit to classify certain classes corresponding to the input vector. In detail, the RF classifier formulation is presented as follows [18–20, 36, 41–43]:

(3) $$\begin{array}{c} \left\{h\left(x,\theta_{k}\right),\;k=1,2,\ldots \right\}, \end{array}$$

where *h* is the result of the random forest classification; *x* is the input sample; and *θ*_*k*_ is the random vector sample as a class in the random forest classification.

### 2.5. Support Vector Machine (SVM) Classifier

The SVM classifier is also a digitally supervised classification approach that is based on the principle of structural risk minimisation and statistical learning to determine the location of boundaries in order to obtain an optimal class separation. It is usually used for pattern classification and nonlinear regression. To be linearly separable, SVM will select a linear decision boundary that leaves the largest margin as the sum of the distances to the hyperplane from the closest point between the two classes. If there are two nonlinear classes, the SVM classifier approach tries to find a hyperplane that maximises margins and minimises a quantity proportional to the number of misclassification errors. In detail, the SVM classifier formulation for linearly inseparable data to find the separating hyperplane is presented as follows [21, 44, 45]:

(4) $$\begin{array}{c} x\in R^{I}⟶\phi \left(x\right)\in R^{H}, \end{array}$$

where *x* is the input data in the input space *I* into a high dimension space *H* and *ϕ*(*x*) is the kernel function.

### 2.6. The Majority Segment-Based Filtering (MaSegFil) Approach

**Figure 4:**
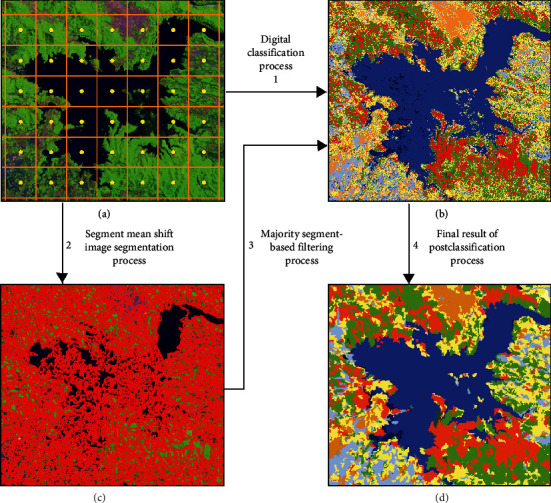
Illustration stages of the MaSegFil approach.

In this study, the MaSegFil approach is proposed as a spatial filter stage in the postclassification of the digital classification results used to classify objects on the earth's surface. In this case, ML, RF, and SVM classifiers are used for the LULC classification. The purpose of using MaSegFil is to reduce pixel noise or error from the digital classification results and to obtain better information on the object classification results. The principles of using the MaSegFil approach are that (a) the area boundary of the class of objects on the earth's surface seen in the satellite image data will be separated by pattern based on the segment mean shift process (segmentation process), (b) the results of the digital classification classifications that have been carried out are used as input to fill in the attribute class of objects in each area segment that has been separated based on its segmentation pattern, (c) the extraction of the attribute value of the digital classification results in each area segment (object segmentation result) is done by taking the majority value or the most dominant object and used as the spatial filter in the area based on the zonal statistics spatial analyst calculation with the type majority, and (d) the final result of the calculation is used as the output of classifying objects at the postclassification stage. In detail, the illustration stages of the MaSegFil approach proposed in this study are presented in Figure 4.

### 2.7. Accuracy Assessment and Comparison of the Classification Performance Results

Accuracy assessment was made to evaluate the results of the digital classifications generated from the ML, RF, and SVM classifiers, together with the optimisation results from using the MaSegFil approach for the postclassification stage as a spatial filter, as proposed in this study. A confusion matrix was used to evaluate the accuracy assessment procedure, which took into account user accuracy, producer accuracy, kappa, and overall accuracy [36, 42, 45–47].

## 3. Results

ML, RF, and SVM classifiers were used as an approach to classify LULC classes, 11 of which were used in the study (Table 1). The training sample and reference map were produced referring to the annual mosaic image data of SPOT 6/7 from 2019, with a GIF arrangement with a size of 2 km × 2 km. Furthermore, the centre point of each GIF block was buffered with a distance of 200 m to obtain the polygon area and was used as a location for the training samples and also as a reference for the LULC classes in the study area (Figure 3). Finally, the MaSegFil approach (Figure 4) was used in a spatial filter stage in the postclassification of the digital classification results that are implemented in the LULC classification results from the ML, RF, and SVM classifiers.

### 3.1. LULC Classification Based on the ML Classifier

**Figure 5:**
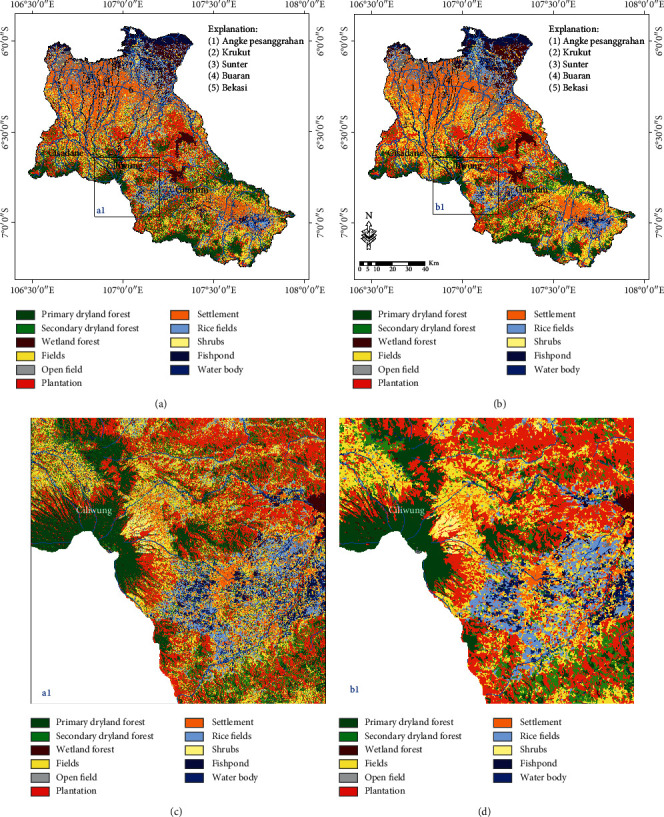
Result of the LULC classification based on the ML classifier: (a, c) process stages without the MaSegFil approach; (b, d) process stages with the MaSegFil approach.

The results of the LULC classification based on the ML classifier are presented in Figure 5, while a comparison before and after the MaSegFil approach stage is presented in Figures 5(a) and 5(b).  Table 2 shows the results of the accuracy assessment of the LULC classification based on the ML classifier without the MaSegFil approach. In addition, the results of the accuracy assessment of the LULC classification based on the ML classifier with the MaSegFil approach are shown in Table 3.

### 3.2. LULC Classification Based on the RF Classifier

**Figure 6:**
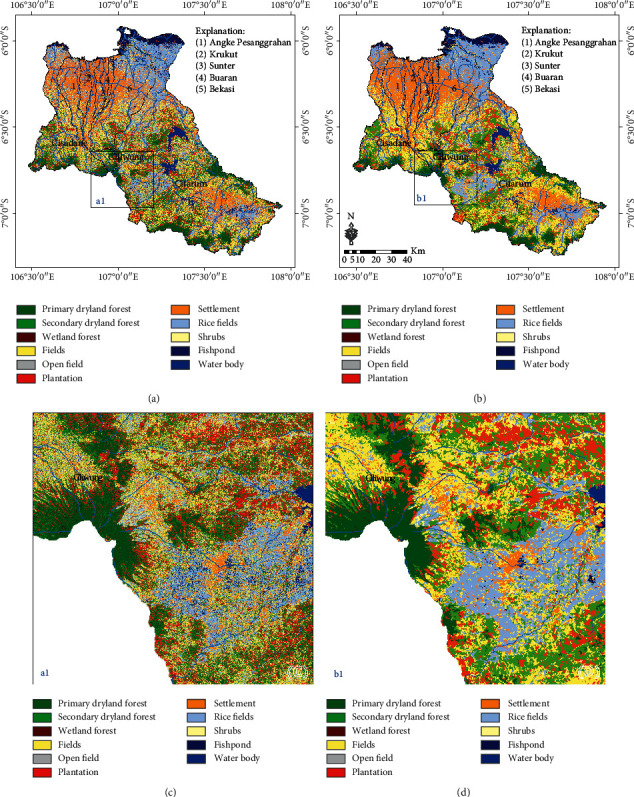
Results of the LULC classification based on the RF classifier: (a, c) process stages without the MaSegFil approach; (b, d) process stages with the MaSegFil approach.

The results of the LULC classification based on the RF classifier are presented in Figure 6, and a comparison of the LULC classification based on the RF classifier before and after the MaSegFil approach stage is presented in Figures 6(a) and 6(b).  Table 4 shows the results of the accuracy assessment of the LULC classification based on the RF classifier without the MaSegFil approach. Moreover, the results of the accuracy assessment of the LULC classification based on the RF classifier with the MaSegFil approach can be seen in Table 5.

### 3.3. LULC Classification Based on the SVM Classifier

**Figure 7:**
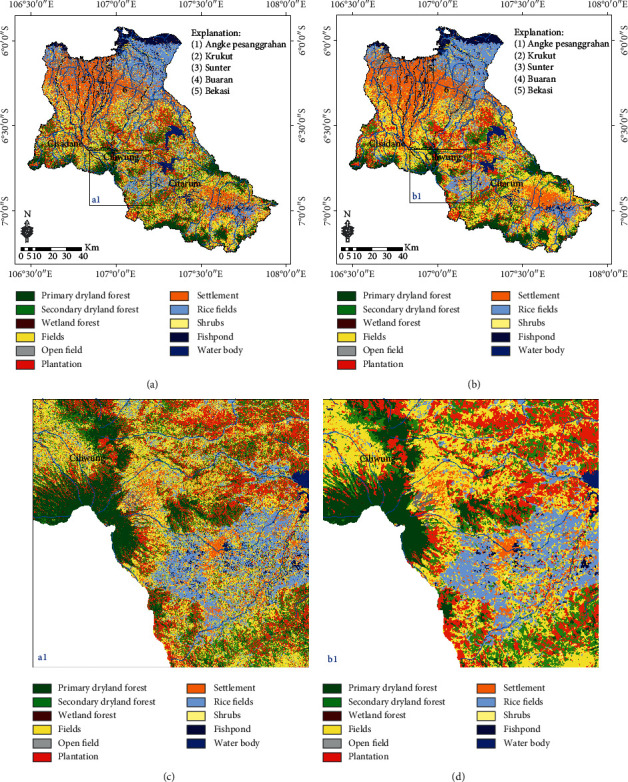
Result of the LULC classification based on the SVM classifier: (a, c) process stages without the MaSegFil approach; (b, d) process stages with the MaSegFil approach.

The results of the LULC classification based on the SVM classifier are presented in Figure 7. In addition, a comparison of the LULC classification based on the SVM machine classifier before and after the MaSegFil approach stage is shown in Figures 7(a) and 7(b).  Table 6 shows the results of the accuracy assessment of the LULC classification based on the SVM classifier without the MaSegFil approach. In addition, the results of the accuracy assessment of the LULC classification based on the SVM classifier with the MaSegFil approach are presented in Table 7.

## 4. Discussion

In this study, we have improved the accuracy of the LULC classification based on the mosaic cloud-free Landsat 8 satellite imagery that can be obtained from GEE, and its popular method for filling gaps in cloudy images using median metrics or the temporal aggregation method [36]. ML, RF, and SVM, which have been widely used for image classification [36, 39, 40, 42, 43], were employed as methods to classify LULC in the study area. We chose the Citarum, Ciliwung, and Cisadane watersheds as test case study areas to be able to understand and implement the proposed method; these areas are included in the 15 national priority watersheds in Indonesia. The accuracy of the assessment results based on reference data from the use of the three methods shows that the overall accuracy and kappa values for the ML classifier for the LULC classification in the study area were 73.60% and 0.684; for RF, they were 77.70% and 0.731; and for SVM, they were 77.5% and 0.730. Spatially, the classification results are shown in Figures 5(a)–5(c), 6(a)–6(c), and 7(a)–7(c). The accuracy of the assessment results for RF and SVM is similar; it has been reported by Rana and Venkata Suryanarayana [45] and Phan et al. [36] that RF and SVM are the latest developments in the computational aspect of image classification and can minimise errors in classification, making them superior to parametric classifiers such as ML.

The results of the LULC classification using the three classifiers still contain pixel noise, which affects the accuracy and quality of LULC information [28, 31–33]. To overcome this obstacle, the MaSegFil approach was proposed as a spatial filter stage in the postclassification, which is used to classify objects on the earth's surface based on digital classification results. The results of the assessment accuracy calculations based on reference data from the use of the MaSegFil approach in the three methods show that the overall accuracy and kappa values for the ML, RF, and SVM classifiers are 81.70% and 0.779, 85.20% and 0.821, and 84.30% and 0.810, respectively. The results from using the MaSegFil approach as a spatial filter stage in the postclassification are shown in Figures 5(b)–5(d), 6(b)–6(d), and 7(b)–7(d).

The highest overall accuracy in relation to the Ciliwung, Citarum, and Cisadane watersheds without using the MaSegFil approach was obtained using the RF classifier, with an overall accuracy of 77.7% and kappa of 0.731, slightly different from the use of the SVM classifier, which had an overall accuracy of 77.5% and kappa of 0.730. The MaSegFil approach utilises segments formed in the segmentation process as the boundary of a class area. When several LULC classes appear in a segment, the most dominant class within it will become the class for the segment area, and the classes that are not dominant will be eliminated or replaced by the most dominant one. This principle is useful because the classification results will usually form small noise classes in a homogeneous class, so it is necessary to eliminate the noise to improve the accuracy. The MaSegFil applied shows improved overall accuracy in all three classifier methods, ranging from 6.8% to 8.1%. The most significant improvement in accuracy occurred using the ML classifier, which rose from 73.6% to 81.7%. At the same time, the highest overall accuracy after implementing the MaSegFil approach occurred when using the RF classifier, at 85.2%. The user accuracy of all classes using all classification methods improved, but the producer accuracy did not improve in all cases, with the accuracy of several classes decreasing.

**Figure 8:**
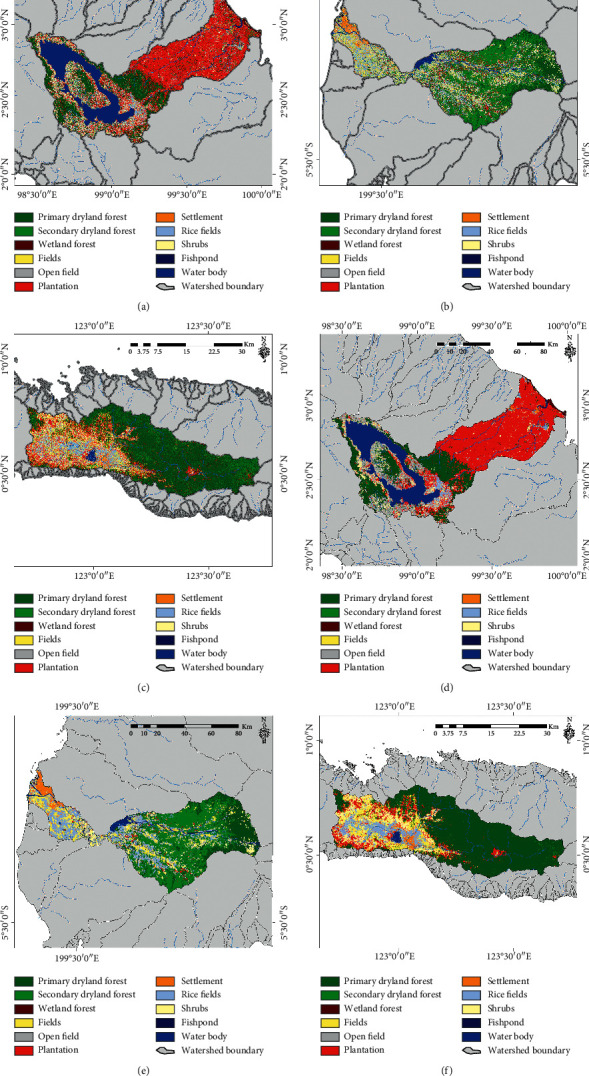
Results of the LULC classification based on the RF classifier: (a, d) Asahan Toba watershed; (b, e) Jeneberang watershed; (c, f) Limboto watershed; process stage without the MaSegFil approach (a–c); process stage with the MaSegFil approach (d–f).

**Figure 9:**
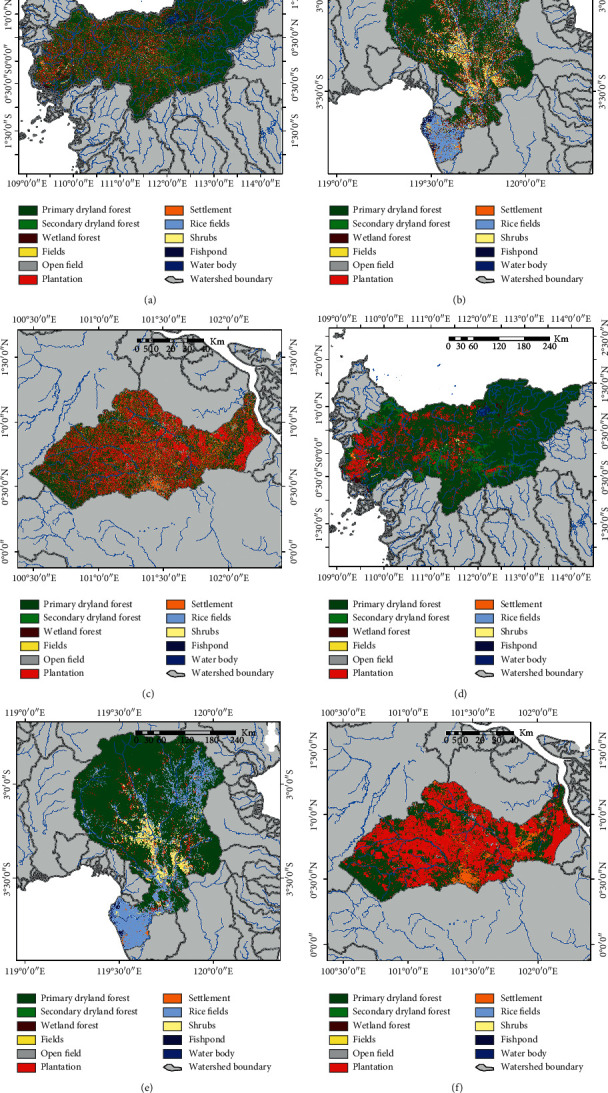
Results of the LULC classification based on the RF classifier: (a, d) Kapuas watershed; (b, e) Saddang watershed; (c, f) Siak watershed; process stage without the MaSegFil approach (a–c); process stage with the MaSegFil approach (d–f).

**Figure 10:**
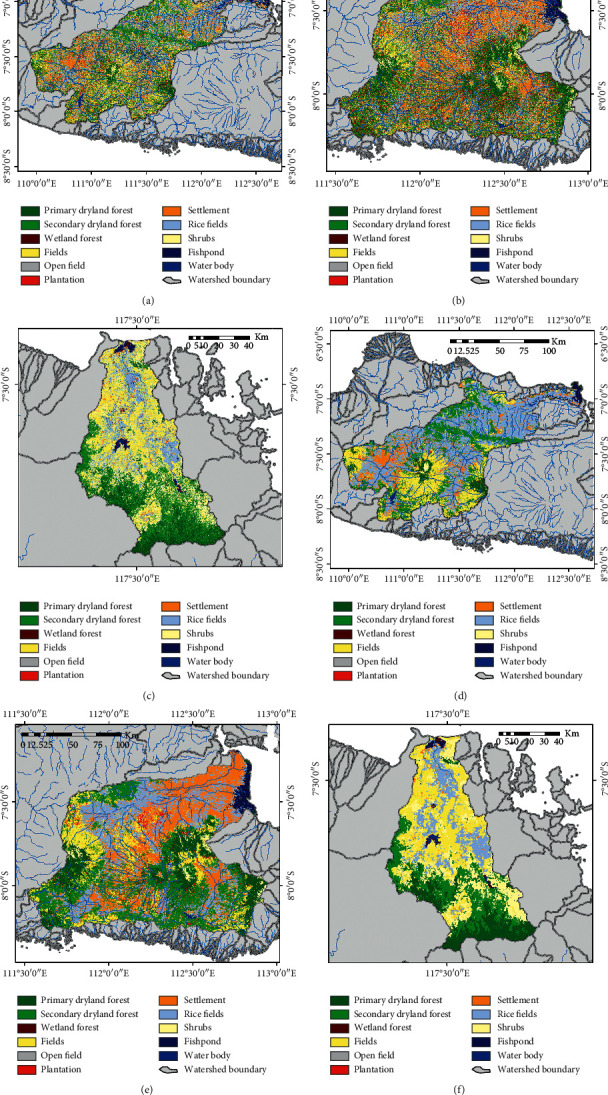
Results of the LULC classification based on the RF classifier: (a, d) Bengawan Solo watershed; (b, e) Brantas watershed; (c, f) Moyo watershed; process stage without the MaSegFil approach (a–c); process stage with the MaSegFil approach (d–f).

**Figure 11:**
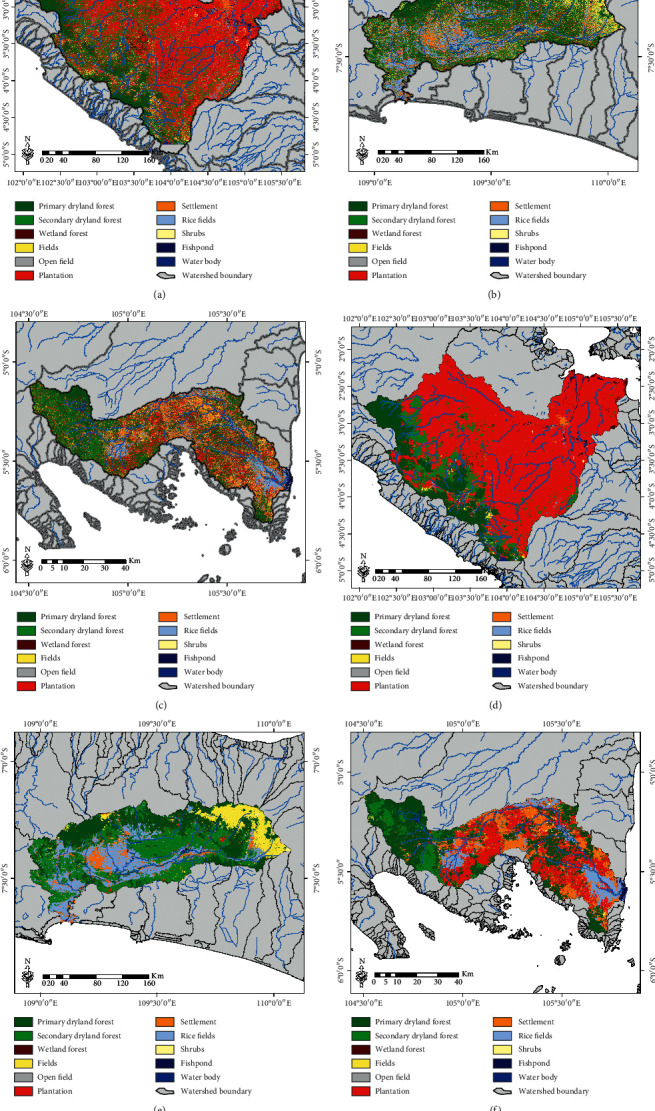
Results of the LULC classification based on the RF classifier: (a, d) Musi watershed; (b, e) Serayu watershed; (c, f) Sekampung watershed; process stage without the MaSegFil approach (a–c); process stage with the MaSegFil approach (d–f).

**Figure 12:**
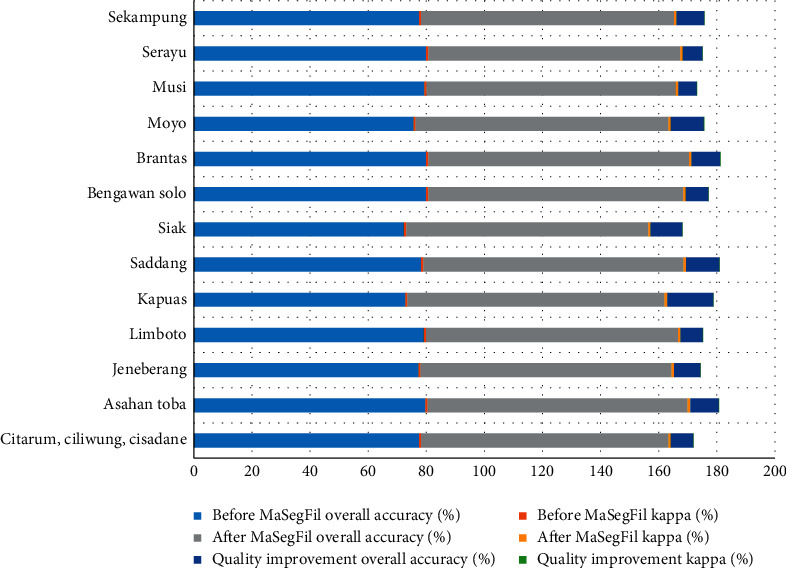
Comparison of the LULC classification accuracy assessment based on the RF classifier before and after using the MaSegFil approach.

For more comprehensive applications, the method that has the best accuracy RF classifier was applied to several national priority watershed locations in Indonesia, with a comparison made of conditions before and after the spatial filter process was conducted using the MaSegFil approach (Figures 8–11). Based on Table 8 and Figure 12, the results show that the use of the MaSegFil approach in the priority watersheds to classify LULC had a variation in overall accuracy ranging from 83.28% to 89.76% and an improvement in accuracy from 6.41% to 15.83%.

In this study, mosaic cloud-free Landsat 8 satellite imagery data were used for the input data to perform the classification. The data applied the median pixel value based on the filter date data as input. The quality and the accuracy will be different if the data used are from single date Landsat 8 data. The use of a single Landsat 8 image is not always possible because several watersheds need more than one path to cover all of their areas, which is not the case in one single date image. Seasonal variations are not considered for the input data, as this would be more challenging because of the varying LULC classes in different conditions. Other optical sensors have yet to be tested, but hypothetically the method would also improve classification accuracy.

## 5. Conclusion

Improvement in the accuracy of the postclassification of LULC is important in order to meet the need for the rapid mapping of such information. This study has proposed the MaSegFil approach, which can be used for spatial filters of supervised digital classification results. Three digital classification approaches (ML, RF, and SVM) were applied to test the improvement in the accuracy of LULC postclassification using the MaSegFil approach. The use of a single Landsat 8 image is not always possible because several watersheds need more than one path to cover all of their areas, which cannot be obtained from one single date image. Mosaic cloud-free Landsat 8 satellite imagery data were used for the input data to make the classification. The data applied the median pixel value based on the filter date data used as input for the LULC classification in the study area. Assessment of the accuracy based on the reference data was made to compare the postclassification results before and after the addition of the MaSegFil approach. The results show that, before applying the MaSegFil approach, the results of the ML, RF, and SVM classifications obtained accuracy were 73.6%, 77.7%, and 77.5%, respectively. However, the MaSegFil approach can reduce pixel noise from the ML, RF, and SVM classifications, with an increase in accuracy of 81.7%, 85.2%, and 84.3%, respectively. Furthermore, the method that has the best accuracy RF classifier was applied to several national priority watershed locations in Indonesia, with a comparison of conditions before and after the spatial filter process was applied using the MaSegFil approach. The results show that the use of the MaSegFil approach implemented on several national priority watersheds in Indonesia to classify LULC had a variation in overall accuracy ranging from 83.28% to 89.76% and an improvement in accuracy from 6.41% to 15.83%. The results of the study can be used to support the acceleration of medium-scale mapping at 1 : 50,000–1 : 100,000, which currently is often performed manually by digitizing on-screen. The development and application of the next method become input for future research in the use of other optical image data that have a higher spatial resolution, such as Sentinel-2, SPOT 6/7, or Pleiades.

## Figures and Tables

**Table 1:** Class for the LULC classification used in this study, referring to the National Standardization Agency for Indonesia [37].

ID	LULC class	Description
1	Primary dryland forest	Forests that grow on dry land habitats, which can be lowland forest, hills, mountains, high plains, or tropical forests that have not experienced human intervention.
2	Secondary dryland forest	Forests that grow on dry land habitats, which can be lowland forest, hills, mountains, high plains, or tropical forests that have undergone human intervention.
3	Wetland forest	Forests that grow in wetland habitats, such as swamps (brackish, peat). Wetland areas have lowland characteristics that extend along the coast, low elevation, and are influenced by tides and other seawater.
4	Fields	Areas used for agricultural activities with the type of crops in the dry land.
5	Rice fields	Agricultural areas and waterlogged or given water with irrigation technology, rain, valleys, or tides characterised by a pattern of ridges, with the planting of short-lived Canaan food (rice).
6	Settlements	Areas of land used as a residential environment and places for activities that support life.
7	Open fields	Open land without cover that is natural, seminatural, or artificial.
8	Plantations	Land used for agricultural activities without replacement crop for two years. Harvests usually take place after a year or more.
9	Shrubs	Dryland areas that have been overgrown with a variety of heterogeneous and homogeneous natural vegetation with sparse to dense density. The area is naturally dominated by low vegetation.
10	Fishponds	Land for fishing or salting activities that appear with a bund pattern around the coast.
11	Water bodies	All types of water areas, such as seas, rivers, lakes, or reservoirs.

**Table 2:** Results of the accuracy assessment for the LULC classification based on the ML classifier without the MaSegFil approach.

LULC class	C_0	C_1	C_2	C_3	C_4	C_5	C_6	C_7	C_8	C_9	C_10	Total	User accuracy	Kappa
C_0	105	22	2	0	0	2	0	0	0	0	0	131	0.802	0.000
C_1	32	199	27	0	0	10	0	0	1	0	0	269	0.740	0.000
C_2	24	19	64	0	0	4	25	21	19	0	0	176	0.364	0.000
C_3	11	0	1	5	0	0	37	1	24	0	0	79	0.063	0.000
C_4	3	5	1	0	3	0	1	11	0	2	0	26	0.115	0.000
C_5	27	49	22	0	0	33	2	1	6	0	0	140	0.236	0.000
C_6	12	0	2	0	0	0	551	6	12	0	0	583	0.945	0.000
C_7	1	0	2	0	0	0	31	357	6	1	0	398	0.897	0.000
C_8	13	0	12	0	0	1	25	8	145	0	0	204	0.711	0.000
C_9	0	0	0	0	0	0	0	4	0	59	5	68	0.868	0.000
C_10	0	0	0	0	0	0	0	0	0	8	45	53	0.849	0.000
Total	228	294	133	5	3	50	672	409	213	70	50	2127	0.000	0.000
Procedure accuracy	0.461	0.677	0.481	1.000	1.000	0.660	0.820	0.873	0.681	0.843	0.900	0.000	0.736	0.000
Kappa	0.000	0.000	0.000	0.000	0.000	0.000	0.000	0.000	0.000	0.000	0.000	0.000	0.000	0.684

C_0: primary dryland forest; C_1: secondary dryland forest; C_2: fields; C_3: open field; C_4: wetland forest; C_5: plantation; C_6: settlement; C_7: rice fields; C_8: shrubs; C_9: fishpond; C_10: water body.

**Table 3:** Result of the accuracy assessment for the LULC classification based on the ML classifier with the MaSegFil approach.

LULC class	C_0	C_1	C_2	C_3	C_4	C_5	C_6	C_7	C_8	C_9	C_10	Total	User accuracy	Kappa
C_0	109	1	0	0	0	1	0	0	0	0	0	111	0.982	0.000
C_1	30	261	28	0	0	9	0	0	0	0	0	328	0.796	0.000
C_2	23	4	79	0	0	2	23	21	4	0	0	156	0.506	0.000
C_3	14	0	1	4	0	0	21	1	20	0	0	61	0.066	0.000
C_4	0	2	3	0	3	0	0	4	0	2	0	14	0.214	0.000
C_5	28	26	13	0	0	38	0	1	4	0	0	110	0.345	0.000
C_6	10	0	4	1	0	0	589	10	0	0	0	614	0.959	0.000
C_7	4	0	0	0	0	0	18	365	1	3	0	391	0.934	0.000
C_8	10	0	5	0	0	0	20	5	184	0	0	224	0.821	0.000
C_9	0	0	0	0	0	0	1	2	0	60	4	67	0.896	0.000
C_10	0	0	0	0	0	0	0	0	0	5	46	51	0.902	0.000
Total	228	294	133	5	3	50	672	409	213	70	50	2127	0.000	0.000
Procedure accuracy	0.478	0.888	0.594	0.800	1.000	0.760	0.876	0.892	0.864	0.857	0.920	0.000	0.817	0.000
Kappa	0.000	0.000	0.000	0.000	0.000	0.000	0.000	0.000	0.000	0.000	0.000	0.000	0.000	0.779

C_0: primary dryland forest; C_1: secondary dryland forest; C_2: fields; C_3: open field; C_4: wetland forest; C_5: plantation; C_6: settlement; C_7: rice fields; C_8: shrubs; C_9: fishpond; C_10: water body.

**Table 4:** Result of the accuracy assessment for the LULC classification based on the RF classifier without the MaSegFil approach.

LULC class	C_0	C_1	C_2	C_3	C_4	C_5	C_6	C_7	C_8	C_9	C_10	Total	User accuracy	Kappa
C_0	102	18	2	0	0	0	0	0	0	0	0	122	0.836	0.000
C_1	40	198	21	0	0	7	1	0	0	0	0	267	0.742	0.000
C_2	30	33	93	0	0	3	24	17	24	0	0	224	0.415	0.000
C_3	4	0	1	4	0	0	14	0	0	0	0	23	0.174	0.000
C_4	1	0	0	0	3	0	0	0	0	1	0	5	0.600	0.000
C_5	16	45	9	0	0	40	1	0	2	0	0	113	0.354	0.000
C_6	13	0	1	1	0	0	569	12	9	0	0	605	0.940	0.000
C_7	1	0	3	0	0	0	23	360	6	1	1	395	0.911	0.000
C_8	21	0	3	0	0	0	38	10	172	0	0	244	0.705	0.000
C_9	0	0	0	0	0	0	2	10	0	66	3	81	0.815	0.000
C_10	0	0	0	0	0	0	0	0	0	2	46	48	0.958	0.000
Total	228	294	133	5	3	50	672	409	213	70	50	2127	0.000	0.000
Procedure accuracy	0.447	0.673	0.699	0.800	1.000	0.800	0.847	0.880	0.808	0.943	0.920	0.000	0.777	0.000
Kappa	0.000	0.000	0.000	0.000	0.000	0.000	0.000	0.000	0.000	0.000	0.000	0.000	0.000	0.731

C_0: primary dryland forest; C_1: secondary dryland forest; C_2: fields; C_3: open field; C_4: wetland forest; C_5: plantation; C_6: settlement; C_7: rice fields; C_8: shrubs; C_9: fishpond; C_10: water body.

**Table 5:** Results of the accuracy assessment for the LULC classification based on the RF classifier with the MaSegFil approach.

LULC class	C_0	C_1	C_2	C_3	C_4	C_5	C_6	C_7	C_8	C_9	C_10	Total	User accuracy	Kappa
C_0	108	0	1	0	0	1	0	0	0	0	0	110	0.982	0.000
C_1	27	268	21	0	0	8	0	0	0	0	0	324	0.827	0.000
C_2	31	9	99	0	0	2	24	17	4	0	0	186	0.532	0.000
C_3	3	0	0	4	0	0	4	0	0	0	0	11	0.364	0.000
C_4	0	0	0	0	3	0	0	0	0	0	0	3	1.000	0.000
C_5	24	17	4	0	0	39	0	0	0	0	0	84	0.464	0.000
C_6	14	0	2	1	0	0	599	9	0	0	0	625	0.958	0.000
C_7	3	0	1	0	0	0	14	374	0	4	0	396	0.944	0.000
C_8	17	0	5	0	0	0	29	7	209	0	0	267	0.783	0.000
C_9	1	0	0	0	0	0	2	2	0	65	5	75	0.867	0.000
C_10	0	0	0	0	0	0	0	0	0	1	45	46	0.978	0.000
Total	228	294	133	5	3	50	672	409	213	70	50	2127	0.000	0.000
Procedure accuracy	0.474	0.912	0.744	0.800	1.000	0.780	0.891	0.914	0.981	0.929	0.900	0.000	0.852	0.000
Kappa	0.000	0.000	0.000	0.000	0.000	0.000	0.000	0.000	0.000	0.000	0.000	0.000	0.000	0.821

C_0: primary dryland forest; C_1: secondary dryland forest; C_2: fields; C_3: open field; C_4: wetland forest; C_5: plantation; C_6: settlement; C_7: rice fields; C_8: shrubs; C_9: fishpond; C_10: water body.

**Table 6:** Result of the accuracy assessment for the LULC classification based on the SVM classifier without the MaSegFil approach.

LULC class	C_0	C_1	C_2	C_3	C_4	C_5	C_6	C_7	C_8	C_9	C_10	Total	User accuracy	Kappa
C_0	101	14	1	0	0	1	0	0	0	0	0	117	0.863	0.000
C_1	39	227	25	0	0	9	0	0	1	0	0	301	0.754	0.000
C_2	29	25	82	0	0	4	28	23	19	0	0	210	0.390	0.000
C_3	6	0	1	4	0	0	24	0	3	0	0	38	0.105	0.000
C_4	3	0	0	0	3	0	1	3	0	1	0	11	0.273	0.000
C_5	17	28	9	0	0	36	1	4	4	0	0	99	0.364	0.000
C_6	10	0	1	1	0	0	555	6	8	0	0	581	0.955	0.000
C_7	1	0	3	0	0	0	26	360	7	1	1	399	0.902	0.000
C_8	22	0	11	0	0	0	37	8	171	0	0	249	0.687	0.000
C_9	0	0	0	0	0	0	0	5	0	66	5	76	0.868	0.000
C_10	0	0	0	0	0	0	0	0	0	2	44	46	0.957	0.000
Total	228	294	133	5	3	50	672	409	213	70	50	2127	0.000	0.000
Procedure accuracy	0.443	0.772	0.617	0.800	1.000	0.720	0.826	0.880	0.803	0.943	0.880	0.000	0.775	0.000
Kappa	0.000	0.000	0.000	0.000	0.000	0.000	0.000	0.000	0.000	0.000	0.000	0.000	0.000	0.730

C_0: primary dryland forest; C_1: secondary dryland forest; C_2: fields; C_3: open field; C_4: wetland forest; C_5: plantation; C_6: settlement; C_7: rice fields; C_8: shrubs; C_9: fishpond; C_10: water body.

**Table 7:** Results of the accuracy assessment for the LULC classification based on the SVM classifier with the MaSegFil approach.

LULC class	C_0	C_1	C_2	C_3	C_4	C_5	C_6	C_7	C_8	C_9	C_10	Total	User accuracy	Kappa
C_0	108	0	0	0	0	1	0	0	0	0	0	109	0.991	0.000
C_1	36	277	27	0	0	14	0	0	0	0	0	354	0.782	0.000
C_2	26	5	90	0	0	3	21	19	3	0	0	167	0.539	0.000
C_3	7	0	0	4	0	0	13	0	0	0	0	24	0.167	0.000
C_4	0	0	0	0	3	0	0	1	0	0	0	4	0.750	0.000
C_5	19	12	7	0	0	32	0	1	0	0	0	71	0.451	0.000
C_6	10	0	3	1	0	0	590	8	0	0	0	612	0.964	0.000
C_7	4	0	1	0	0	0	18	370	0	4	0	397	0.932	0.000
C_8	18	0	5	0	0	0	29	8	210	0	0	270	0.778	0.000
C_9	0	0	0	0	0	0	1	2	0	65	5	73	0.890	0.000
C_10	0	0	0	0	0	0	0	0	0	1	45	46	0.978	0.000
Total	228	294	133	5	3	50	672	409	213	70	50	2127	0.000	0.000
Procedure accuracy	0.474	0.942	0.677	0.800	1.000	0.640	0.878	0.905	0.986	0.929	0.900	0.000	0.843	0.000
Kappa	0.000	0.000	0.000	0.000	0.000	0.000	0.000	0.000	0.000	0.000	0.000	0.000	0.000	0.810

C_0: primary dryland forest; C_1: secondary dryland forest; C_2: fields; C_3: open field; C_4: wetland forest; C_5: plantation; C_6: settlement; C_7: rice fields; C_8: shrubs; C_9: fishpond; C_10: water body.

**Table 8:** Results of the LULC classification accuracy assessment based on the RF classifier before and after using the MaSegFil approach.

Watershed	Before MaSegFil		After MaSegFil		Quality improvement	
	Overall accuracy (%)	Kappa	Overall accuracy (%)	Kappa	Overall accuracy (%)	Kappa
Citarum, Ciliwung, Cisadane	77.50	0.731	85.20	0.821	7.70	0.090
Asahan Toba	79.66	0.785	89.53	0.879	9.87	0.094
Jeneberang	77.31	0.765	86.41	0.854	9.10	0.089
Limboto	79.14	0.777	86.80	0.814	7.66	0.037
Kapuas	72.78	0.722	88.61	0.866	15.83	0.144
Saddang	78.19	0.750	89.65	0.889	11.46	0.139
Siak	72.35	0.794	83.28	0.808	10.93	0.014
Bengawan Solo	79.93	0.786	87.76	0.865	7.83	0.079
Brantas	79.96	0.780	89.76	0.885	9.80	0.104
Moyo	75.66	0.706	87.02	0.860	11.36	0.154
Musi	79.35	0.792	85.76	0.844	6.41	0.052
Serayu	79.96	0.780	86.76	0.855	6.80	0.074
Sekampung	77.49	0.762	87.05	0.865	9.56	0.103

## Data Availability

The data used to support the findings of this study are available from the corresponding author upon request.
